# Are psychological measures and actuarial data equally effective in discriminating among the prison population? Analysis by crimes

**DOI:** 10.1371/journal.pone.0198251

**Published:** 2018-06-06

**Authors:** Carlos Burneo-Garcés, Manuel Fernández-Alcántara, Agar Marín-Morales, Miguel Pérez-García

**Affiliations:** 1 Department of Personality, Assessment and Psychological Treatment, University of Granada (UGR), Granada, Spain; 2 Mind, Brain and Behavior Research Center, University of Granada (CIMCYC-UGR), Granada, Spain; 3 University of Otavalo, Otavalo, Ecuador; 4 Department of Health Psychology, University of Alicante, Alicante, Spain; 5 Mental Health Networking Biomedical Research Center (CIBERSAM), Granada, Spain; Universidad de Extremadura, SPAIN

## Abstract

The ability of a wide range of psychological and actuarial measures to characterize crimes in the prison population has not yet been compared in a single study. Our main objective was to determine if the discriminant capacity of psychological measures (PM) and actuarial data (AD) varies according to the crime. An Ecuadorian sample of 576 men convicted of Robbery, Murder, Rape and Drug Possession crimes was evaluated through an *ad hoc* questionnaire, prison files and the Spanish adaptation of the Personality Assessment Inventory. Discriminant analysis was used to establish, for each crime, the discriminant capacity and the classification accuracy of a model composed of AD (socio-demographic and judicial measures) and a second model incorporating PM. The AD showed a superior discriminant capacity, whilst the contribution of both types of measures varied according to the crime. The PM generated some increase in the correct classification percentages for Murder, Rape and Drug Possession, but their contribution was zero for the crime of Robbery. Specific profiles of each crime were obtained from the strongest significant correlations between the value of each explanatory variable and the probability of belonging to the crime. The AD model is more robust when these four crimes are characterized. The contribution of AD and PM depends on the crime, and the inclusion of PM in actuarial models moderately optimizes the classification accuracy of Murder, Rape, and Drug Possession crimes.

## Introduction

In-prison violence has attracted the attention of researchers because of its implications for processes related to intervention and security [1–3]. An example of this is the importance given to the identification of variables that are strongly associated with a variety of forms of aggression, from which models have been designed to predict recidivism [4]. In fact, data from different individual facets (e.g., socio-demographic characteristics, criminal history, personality, psychopathology, interpersonal relationships, interaction with the environment) is frequently used to analyze violent behavior in correctional settings. However, the exclusive or predominant presence of a class of measures in a study often depends on establishing their usefulness *a priori*.

This trend can be seen, for example, in the systematic review conducted by Schenk and Fremouw [5] on individual characteristics related to prison violence in English-speaking populations. The analyzed variables were divided into three categories: demographic, criminal history, and psychological. Studies revealed that the likelihood of being involved in violent acts is inversely related to age and education [6,7]. In addition, having a more extensive arrest history [8,9], being convicted of crimes against property [7,10], serving shorter sentences [8,11] and belonging to a gang [12,13] correlates positively with a greater occurrence of violent behavior in prison. With regard to psychological variables, the conclusions indicate that individuals with more aggressive tendencies, a history of severe mental disorders, symptoms of confusion, high self-esteem (for white inmates) and less social support are more likely to be involved in acts of violence [14–18]. Although only a few studies that provide data on psychological measures met the inclusion criteria, the findings provided by these measures were not as consistent as those provided by the other variables. Finally, one of the main methodological weaknesses identified is the lack of integration of variables of a different nature within the same study, which would serve to identify the contribution or relative importance of each one.

This limitation also affects similar and complementary subjects of study related to violence in the prison population, as in the case of detecting measures that differentiate—within prison and with sufficient accuracy—individuals who have committed crimes with and without violence. This is due to the fact that it is not usual to analyze several crimes within the same study, let alone use a wide range of measures (e.g., socio-demographic, judicial and psychological) for the aforementioned purpose. Moreover, to the best of our knowledge, there is no study that meets all of these characteristics. Even among studies analyzing the same type of offenders, the existing methodological diversity hinders the acquisition of consistent data that lead to the construction of a clear and distinct profile [19,20]. Consequently, greater methodological rigor could have an impact on obtaining reliable and useful data on related study topics within prisons (e.g., defining characteristics of offender populations, violence, prediction of recidivism risk).

This conclusion is particularly relevant to psychological variables [5], which can play a role in intervention, risk assessment, and risk reduction [4,21,22]. The latter authors have defined four types of risk factor for violence: (a) Fixed marker, (b) Variable marker, (c) Variable risk factor, and (d) Causal risk factor. Fixed markers (e.g., Male gender) cannot be modified; Variable markers (e.g., young age) can change over time but cannot be modified through intervention, while variable risk factors (e.g., unemployed) and causal risk factors (e.g., substance abuse) can be modified through intervention. Moreover, the changes that the intervention generates in the causal risk factors can have repercussions for the reduction of recidivism, a capacity that doubles their utility. Therefore, the need to obtain psychological measures with sufficient psychometric properties is evident, a quality that may favor its inclusion in studies of this nature [23].

It is important to note that a correspondence between the discriminative capacity of a variable and its predictive capacity for recidivism should not necessarily be expected. In fact, not all the distinguishing factors of individuals who have committed a crime are strong predictors of their recurrence [24]. In addition, the same risk factors may appear as the main predictors of general and violent recidivism in some populations of offenders [24]. This suggests the need to improve the accuracy of the instruments used for this purpose [25], an objective that involves refining the analyses at different levels of complexity. One conclusion that sums up the previous considerations is that certain shortcomings could be solved with methodologically exhaustive and rigorous approaches that explore the distinctive characteristics of several types of offenders within the same study and that employ a broad set of psychological measures (e.g., anxiety, schizophrenia, substance abuse, aggression, stress, social support) and actuarial data (e.g., age, marital status, education, employment status before entering prison, prior prison terms). Such designs would be of multiple use as they would make it possible to ascertain the effectiveness of different types of measures to accurately characterize the prison population in terms of crimes and, by extension, to identify the measures that have the greatest discriminatory power for each of them.

Therefore, the main objective of this study was to determine whether the discriminatory capacity of psychological measures (PM) and actuarial data (AD)—including socio-demographic measures (SDM) and judicial measures (JM)—varies according to the crime. For this, a large sample of males convicted of Robbery, Murder, Rape, and Drug Possession were employed. These crimes belong to the four crime categories (Property offenses, Intentional homicide, Other violent offenses, and Drug-related offenses), which are those most frequently reported in the international prison population [26]. On the basis of the above review, two hypotheses are proposed: (a) That the AD will show a greater discriminatory capacity in the four crimes of study, and (b) that each crime will present a specific profile.

## Material and methods

### Sample

A set of 811 individuals was selected by random sampling, using the official list of male sentenced prisoners from the Regional Guayas Social Rehabilitation Center (CRSRG) and the Guayaquil Social Rehabilitation Center (CRSG). These adult male prisons, which house approximately 9,000 inmates, are located in Guayaquil, Ecuador. The prison population in this country is estimated to be around 26,000 [27]. For the purpose of this study, individuals were grouped according to whether they were serving sentences for the four most frequent crimes [26]. Thus, the study sample was composed of 576 male sentenced prisoners aged 19–74 years (*M* = 35.58; *SD* = 10.13) for the crimes of Robbery (*N* = 210; 36.5%), Murder (*N* = 158; 27.4%), Rape (*N* = 108; 18.8%) and Drug Possession (*N* = 100; 17.4%). The distribution of the sample by centers was as follows: CRSRG = 371 (64.4%) and CRSG = 205 (35.6%).

### Inclusion and exclusion criteria

The inclusion criteria were: (a) serving a sentence in either CRSRG or CRSG, and (b) participating voluntarily in the study. The exclusion criteria were: (a) having insufficient knowledge of the Spanish language, (b) being in an inadequate physical or mental state to complete the questionnaires, and (c) having an attitude that precludes the development of evaluation. The exclusion criteria were taken into account from the first contact with the inmate until the end of the evaluation. Thus, the proportion of excluded participants (5%) was composed of individuals that did not declare interest in the study, had difficulties with language comprehension, or, upon beginning the evaluation, showed misconduct or lack of motivation to continue the study. For those cases, the information provided by the participants was deleted immediately. The excluded participants had the same characteristics as the 576 individuals who had satisfactorily completed the evaluation.

### Instruments and measures

The main criteria applied for the selection of PM and AD were psychometric utility and frequency of use in correctional settings [5,28–30]. The measures included in this study were from three sources: (1) an *ad hoc* questionnaire to gather socio-demographic and criminal history, (2) the criminal justice records of both prisons, and (3) the Spanish adaptation of PAI [31].

The PAI is a self-report that measures the effect of thoughts, attitudes, behaviors, facts, and past and present circumstances on the development of symptoms, the characteristics of personality, and the individual’s behavior at the time of evaluation. It is composed of 4 validity scales, 11 clinical scales, 5 scales for treatment consideration, 2 interpersonal relation scales, 31 subscales, and 10 complementary indexes (the content of the 22 scales is non-overlapping). The clinical scales represent the clinical syndromes of the highest significance in diagnostic practice, whereas the scales related to the treatment provide complementary information that could be relevant to a possible intervention. Finally, the interpersonal scales measure the interpersonal relationship style, whereas the complementary indexes can be used to obtain a more precise interpretation of some of the scores. This tool is composed of 344 items that use a Likert scale with four response alternatives: 1 = False, 2 = Slightly True, 3 = Mainly True, and 4 = Very True. Completion of the questionnaire requires fourth-grade reading level and takes 50–60 minutes.

The Spanish adaptation of the PAI has adequate psychometric properties [31]. The median of the Cronbach’s alpha coefficients was .78 for the scales, while for the subscales this was .70. When analyzing temporal consistency, the median of the test-retest correlations obtained was .84 for the scales and .79 for the subscales. In addition, Ortiz-Tallo *et al*. [31] compared the average T scores of the typical sample of the Spanish adaptation with the American scale of the PAI [32] and found differences in effect sizes that were non-significant for 17 of the 21 scales, and small for the remaining 4 scales. They concluded that the results obtained were consistent with those found in the original studies [32,33]. Finally, the PAI has revealed acceptable psychometric properties in the Ecuadorian prison population [34]. These authors reported that the internal structure of the Spanish version of this instrument was consistent with the three invariant component structure described by Hoelzle and Meyer [35] (i.e., general distress, elevated mood, and dominance, and substance abuse and psychopathy), with Cronbach’s alpha coefficients ranging from .49 to .89.

Given the absence of specific norms for Spanish-speaking Latin American populations at the time of evaluation, the Spanish norms were used in the present study. With respect to validity criteria, Ortiz-Tallo *et al*. [31] have indicated two strategies with high sensitivity and specificity to detect random response in general and clinical populations using two validity scales: (1) Inconsistency (ICN) ≥ 75T or Infrequency (INF) ≥ 75T, and (2) ICN ≥ 64T and INF ≥ 60T. However, they also highlighted the limited usefulness of the INF scale in correctional settings since the high scores on this scale appear to be more related to situational characteristics than to a random response pattern. Given these considerations, we preferred to apply the ICN ≥ 75T cut-off point. For the Negative Impression (NIM) and Positive Impression (PIM) validity scales, the ≥ 101T and ≥ 65T cut-off points were taken into account respectively [31]. With the sequential application of the cut-off points ICN ≥ 75T, NIM ≥ 101T and PIM ≥ 65T to the study sample, the lost cases were 59, 46, and 21 respectively. Therefore, 450 participants aged 20–74 years (*M* = 35.10; *SD* = 9.94) were classified as meeting the validity criteria for the current study, and the distribution by crime was as follows: Robbery (*N* = 166; 36.9%), Murder (*N* = 127; 28.2%), Rape (*N* = 82; 18.2%) and Drug Possession (*N* = 75; 16.7%). Finally, the distribution of our sample by center was as follows: CRSRG = 298 (66.2%) and CRSG = 152 (33.8%).

### Categorical and explanatory variables

The crimes of Robbery, Murder, Rape and Drug Possession, classified according to the Organic Integral Criminal Code of the Republic of Ecuador were designated as dependent variables [36]. Regarding the explanatory variables, 9 SDM, 2 JM and 18 PM (11 clinical, 5 related to treatment and 2 of interpersonal relationships) were included in the study. The SDM were represented by: (a) Age, (b) Marital status at the time of evaluation, broken down into Single/Widowed, Common law, Married, and Separated/Divorced, (c) Number of children, (d) Years of education (total years of study), (e) Education (level of education completed), and (f) Employment status (considering any job or professional activity, formal or informal, with a stable and regular income prior to entering prison). In addition, the JM considered were Prior prison terms and Total prison terms. Finally, the PAI provided a wide range of PM through its 11 clinical scales (SOM, ANX, ARD, DEP, MAN, PAR, SCZ, BOR, ANT, ALC, and DRG), 5 scales for treatment consideration (AGG, SUI, STR, NON, and RXR), and 2 interpersonal relation scales (DOM and WRM).

### Procedure

The Undersecretariat of Rehabilitation, Reintegration, and Precautionary Measures for Adults (Ministry of Justice, Human Rights, and Cults of Ecuador) granted the necessary permits. Statistical information and coordination of the study in the centers according to the required security rules were requested from the directors of the two prisons. A team of nine psychologists from the Ministry of Public Health of Ecuador (MSP) conducted the fieldwork between February and April 2015, none of which had any authority or connections within the prison context. In addition, they received training in forensic psychopathology, mental health research, application of the research protocol, and recording the information. Disciplinary rules of prisons, individual characteristics (physical and psychological) of participants, and the time available for the fieldwork, suggested an assessment procedure that was as short and useful as possible. The *ad -hoc* questionnaire was administered immediately after the PAI. In total, the individual evaluation took between 70 and 90 minutes. The participants received the necessary assistance to solve any difficulties caused by the linguistic differences between the Spanish used in Ecuador and that used in the PAI. In terms of the frequency and characteristics of the difficulties encountered during the evaluations, there were no major drawbacks in this area. The present study is part of and uses data from a broader project entitled “Study of the Prevalence of Mental Disorders in Prison Population of Guayaquil".

### Ethics statement

The National Directorate of Primary Healthcare (MSP) reviewed the technical aspects of the study. The Health Coordination Zone 8 (CZ8-S, MSP) managed both the ethics revision and the project approval. The inmates selected by the sampling method were contacted in their pavilion or their security level, where they were given, both individually and in a group, information regarding the characteristics of the study whereupon they could freely decide whether or not to participate in the study. The lack of any kind of benefit in the short, medium, or long-term for their participation in the study was explained, as well as their freedom to leave the study at any time. All individuals signed the Informed Consent Form after listening and reading about the characteristics of the study and the Rights guaranteed to research participants by the Constitution of the Republic of Ecuador [37]. This study followed the ethical principles of the Declaration of Helsinki and the Guidelines for Good Clinical Practice of the European Union.

### Analysis

Descriptive statistics were used to present the sociodemographic, judicial, and psychological characteristics of the participants. The test of equality of means allowed for determining if the groups differ in the explanatory variables selected, while Box’s M test was used to contrast the equality of the covariance matrices of the groups. Once the necessary assumptions had been confirmed, a discriminant analysis was conducted, introducing all explanatory variables simultaneously, into two models: Model 1 = AD; and Model 2 = Model 1 + PM. Regarding PM, the raw scores of the 18 PAI scales were used. This allowed us to evaluate the discriminant capacity of the explanatory variables and the predictive accuracy of the discriminant function of each model. Finally, to compare the relevance of the contribution of all the explanatory variables, the correlation between its value and the probability of belonging to the group was also analyzed. From these results, a profile of each crime was configured with correlations that obtained values *r* >.25 and with a level of significance *p* < .001. These criteria were applied to ensure a reasonable minimum percentage of shared variance. All data were processed using the statistical packages IBM^®^SPSS.22 for Windows [38].

## Results

### Socio-demographic and psychological characteristics

Given that we failed to find any significant statistical differences between the two centers in terms of socio-demographic characteristics, the data were processed as a single sample (Table 1). In the present study, we will provide information only on the subsample (*N* = 450). This is based on the assumption that the application of the validity criteria to the PAI protocol ensures more reliable information. Tables 2 and 3 show the mean and standard deviation of the raw and T scores of the 22 PAI scales for the subsample for each crime.

**Table 1:** Sociodemographic and judicial characteristics of the subsample and the four crimes.

Variable	Subsample *N* = 450	Robbery *N* = 166	Murder *N* = 127	Rape *N* = 82	Drug Possession *N* = 75
	*n* or *M* (% or *SD*)	*n* or *M* (% or *SD*)	*n* or *M* (% or *SD*)	*n* or *M* (% or *SD*)	*n* or *M* (% or *SD*)
Age	35.10 (09.94)	31.09 (07.89)	34.78 (08.19)	39.21 (12.01)	40 (10.43)
Age range:					
18–30 years	183 (40.7)	105(63.3)	43 (33.9)	22 (26.8)	13 (17.3)
31–45 years	199 (44.2)	51 (30.7)	70 (55.1)	35 (42.7)	43 (57.3)
46–60 years	58 (12.9)	10 (06.0)	13 (10.2)	21 (25.6)	14 (18.7)
61–75 years	10 (02.2)	0 (00.0)	1 (00.8)	4 (04.9)	5 (06.7)
Country of origin:					
Ecuador	427 (94.9)	162 (97.6)	121 (95.3)	82 (100)	62 (82.7)
Other countries	23 (05.1)	4 (02.4)	6 (04.7)	0 (00.0)	13 (17.3)
Marital status:					
Single/Widowed	127 (28.2)	56 (33.7)	29 (22.8)	25 (30.5)	17 (22.7)
Common law	224 (49.8)	82 (49.5)	81 (63.8)	31 (37.8)	30 (40.0)
Married	52 (11.6)	14 (08.4)	6 (04.7)	14 (17.1)	18 (24.0)
Separated/Divorced	47 (10.4)	14 (08.4)	11 (08.7)	12 (14.6)	10 (13.3)
Number of children	2.36 (02.18)	1.81 (01.73)	2.61 (02.16)	3.02 (02.61)	2.41 (02.34)
Years of education	8.54 (03.49)	8.46 (03.12)	8.14 (03.41)	8.50 (03.82)	9.43 (03.95)
Education:					
None^a^	74 (16.4)	24 (14.4)	26 (20.5)	12 (14.6)	12 (16.0)
Primary	281 (62.5)	109 (65.7)	76 (59.8)	57 (69.5)	39 (52.0)
Secondary	87 (19.3)	32 (19.3)	24 (18.9)	10 (12.2)	21 (28.0)
Superior	8 (01.8)	1 (00.6)	1 (00.8)	3 (03.7)	3 (04.0)
Employment status:					
Employed 691 (85.2)	390 (86.7)	148 (89.2)	107 (84.3)	75 (91.5)	60 (80.0)
Unemployed	60 (13.3)	18 (10.8)	20 (15.7)	7 (08.5)	15 (20.0)
Prior prison terms:					
No	228 (50.7)	47 (28.3)	75 (59.1)	65 (79.3)	41 (54.7)
Yes	222 (49.3)	119 (71.7)	52 (40.9)	17 (20.7)	34 (45.3)
Total prison terms	1.16 (01.81)	1.9 (02.26)	0.8 (01.29)	0.48 (01.28)	0.88 (01.36)

*Note*. Subsample = PAI profiles that meet the validity criteria for the current study.

^a^This condition does not imply illiteracy.

**Table 2:** Mean and standard deviation of the raw scores of the 22 PAI scales for the subsample and the four crimes.

PAI scale	Subsample *N* = 450	Robbery *N* = 166	Murder *N* = 127	Rape *N* = 82	Drug Possession *N* = 75
	*M* (*SD*)	*M* (*SD*)	*M* (*SD*)	*M* (*SD*)	*M* (*SD*)
**Validity**					
Inconsistency (ICN)	13.84 (04.01)	13.84 (04.12)	13.98 (03.78)	13.87 (04.14)	13.59 (04.07)
Infrequency (INF)	07.63 (02.43)	07.92 (02.47)	07.46 (02.30)	07.57 (02.23)	07.37 (02.71)
Negative Impression (NIM)	05.20 (03.40)	05.64 (03.35)	05.13 (03.25)	04.95 (03.67)	04.60 (03.38)
Positive Impression (PIM)	15.04 (04.54)	14.54 (04.45)	15.03 (04.42)	15.26 (04.91)	15.92 (04.48)
**Clinical**					
Somatic Complaints (SOM)	21.01 (10.41)	20.39 (09.90)	21.78 (10.07)	22.12 (10.94)	19.88 (11.42)
Anxiety (ANX)	24.91 (09.84)	25.04 (09.34)	25.87 (09.44)	24.78 (10.94)	23.13 (10.30)
Anxiety-Related Disorders (ARD)	30.02 (09.07)	30.48 (08.29)	30.55 (08.65)	29.37 (09.96)	28.84 (10.33)
Depression (DEP)	23.58 (09.51)	23.70 (09.16)	24.89 (09.40)	23.60 (09.91)	21.05 (09.71)
Mania (MAN)	30.38 (08.62)	31.43 (08.07)	29.23 (08.77)	30.61(08.56)	29.75 (09.46)
Paranoia (PAR)	31.92 (07.82)	32.81 (07.14)	31.49 (07.91)	30.80 (08.36)	31.88 (08.41)
Schizophrenia (SCZ)	22.85 (08.77)	23.85 (08.86)	22.57 (07.99)	22.63 (09.06)	21.32 (09.38)
Borderline Features (BOR)	27.88 (09.41)	29.10 (09.01)	27.98 (09.19)	26.46 (10.36)	26.53 (09.34)
Antisocial Features (ANT)	24.38 (08.90)	26.90 (08.74)	22.35 (08.51)	23.12 (09.04)	23.63 (08.59)
Alcohol Problems (ALC)	08.23 (07.26)	09.48 (07.60)	06.53 (06.74)	08.76 (07.35)	07.80 (06.76)
Drug Problems (DRG)	08.92 (08.33)	10.60 (08.29)	07.38 (07.88)	07.98 (08.33)	08.85 (08.65)
**Treatment consideration**					
Aggression (AGG)	16.48 (08.56)	18.15 (08.26)	14.97 (08.15)	16.16 (09.01)	15.69 (08.94)
Suicide Ideation (SUI)	04.00 (04.86)	03.60 (04.26)	04.37 (05.32)	05.32 (05.74)	02.80 (03.83)
Stress (STR)	10.58 (03.99)	10.90 (03.75)	10.89 (03.66)	10.32 (04.61)	09.63 (04.24)
Non-Support (NON)	09.85 (03.66)	10.01 (03.51)	09.67 (03.52)	09.76 (03.87)	09.93 (04.04)
Treatment Rejection (RXR)	11.96 (03.84)	11.64 (03.80)	12.17 (03.80)	11.57 (03.73)	12.73 (04.05)
**Interpersonal relations**					
Dominance (DOM)	21.32 (06.28)	21.08 (06.18)	20.98 (06.52)	21.67 (06.25)	22.05 (06.18)
Warmth (WRM)	22.98 (05.68)	22.57 (05.91)	23.50 (05.62)	22.63 (06.02)	23.36 (04.85)

*Note*. Subsample = PAI profiles that meet the validity criteria for the current study.

**Table 3:** Mean and standard deviation of the T scores of the 22 PAI scales for the subsample and the four crimes.

PAI scale	Subsample *N* = 450	Robbery *N* = 166	Murder *N* = 127	Rape *N* = 82	Drug Possession *N* = 75
	*M* (*SD*)	*M* (*SD*)	*M* (*SD*)	*M* (*SD*)	*M* (*SD*)
**Validity**					
Inconsistency (ICN)	57.96 (09.34)	57.87 (09.71)	58.34 (08.47)	58.24 (10.03)	57.19 (09.24)
Infrequency (INF)	71.59 (10.67)	72.58 (10.53)	71.05 (10.27)	71.79 (10.62)	70.08 (11.63)
Negative Impression (NIM)	65.65 (15.02)	67.64 (15.10)	65.39 (14.20)	64.59 (16.19)	62.85 (14.59)
Positive Impression (PIM)	49.39 (09.35)	48.09 (09.19)	49.50 (08.88)	50.10 (10.21)	51.33 (09.26)
**Clinical**					
Somatic Complaints (SOM)	59.74 (10.76)	58.98 (10.32)	60.78 (10.65)	60.94 (10.85)	58.35 (11.65)
Anxiety (ANX)	54.77 (08.88)	54.80 (08.59)	55.80 (08.70)	54.73 (09.37)	52.99 (09.13)
Anxiety-Related Disorders (ARD)	59.10 (08.40)	59.52 (08.07)	59.72 (07.74)	58.51 (08.91)	57.79 (09.55)
Depression (DEP)	59.68 (10.36)	59.69 (10.02)	61.29 (10.41)	59.88 (10.64)	56.71 (10.31)
Mania (MAN)	61.14 (09.31)	62.34 (09.07)	59.87 (09.23)	61.63 (09.12)	60.12 (09.92)
Paranoia (PAR)	62.98 (08.18)	63.84 (07.37)	62.66 (08.27)	61.85 (08.66)	62.87 (09.13)
Schizophrenia (SCZ)	60.84 (10.79)	61.97 (10.88)	60.61 (09.77)	60.83 (11.38)	58.72 (11.44)
Borderline Features (BOR)	58.89 (09.41)	60.05 (09.13)	59.08 (09.05)	57.60 (10.43)	57.40 (09.26)
Antisocial Features (ANT)	64.25 (10.81)	67.53 (10.80)	61.61 (10.04)	62.83 (10.93)	63.01 (10.31)
Alcohol Problems (ALC)	61.81 (17.82)	65.00 (18.82)	57.60 (16.36)	63.04 (17.54)	60.53 (16.99)
Drug Problems (DRG)	61.08 (18.80)	64.91 (18.83)	57.65 (17.86)	59.18 (19.13)	60.45 (18.77)
**Treatment consideration**					
Aggression (AGG)	54.11 (10.94)	56.17 (10.28)	52.09 (10.39)	54.24 (12.25)	52.80 (11.14)
Suicide Ideation (SUI)	54.65 (12.97)	53.55 (11.35)	55.69 (14.13)	57.82 (14.65)	51.87 (11.69)
Stress (STR)	61.89 (10.37)	62.65 (10.12)	62.77 (09.32)	61.24 (11.26)	59.43 (11.32)
Non-Support (NON)	62.76 (10.16)	63.11 (09.64)	62.31 (09.96)	62.56 (10.77)	63.00 (11.47)
Treatment Rejection (RXR)	43.73 (07.61)	42.87 (07.56)	44.40 (07.56)	42.96 (07.33)	45.33 (07.91)
**Interpersonal relations**					
Dominance (DOM)	53.36 (10.46)	52.63 (10.67)	52.77 (10.51)	54.55 (09.90)	54.69 (10.48)
Warmth (WRM)	54.16 (08.64)	53.14 (09.46)	55.36 (07.80)	53.87 (09.31)	54.68 (07.11)

*Note*. Subsample = PAI profiles that meet the validity criteria for the current study.

### Discriminant capacity of explanatory variables

The tests of equality of means yielded the following significant values, in order of minor to major Wilk’s Lambda: Prior prison terms, Age, Total prison terms, ANT, Marital status, ALC, SUI, DRG, and AGG. In addition, DEP revealed a marginally significant value. Therefore, only 4 SDM, 2 JM, and 6 PM showed discriminant capacity (Table 4). Complementarily, the Box’s M test (*p* < .001) confirmed that the covariance matrices were not the same.

**Table 4:** Tests of equality of group means.

Independent variable	Wilk’s Lambda	F	*p*
Age	.868	22.678	.000***
Single/Widowed	.987	1.894	.130
Common law	.961	6.029	.000***
Married	.953	7.346	.000***
Separated/Divorced	.993	1.117	.342
Number of children	.956	6.839	.000***
Years of education	.985	2.212	.086
Education	.989	1.708	.165
Employment status	.987	2.026	.110
Prior prison terms	.858	24.684	.000***
Total prison terms	.896	17.183	.000***
Somatic Complaints (SOM)	.993	1.033	.378
Anxiety (ANX)	.992	1.237	.296
Anxiety-Related Disorders (ARD)	.994	.853	.465
Depression (DEP)	.983	2.606	.051
Mania (MAN)	.988	1.745	.157
Paranoia (PAR)	.991	1.412	.239
Schizophrenia (SCZ)	.990	1.543	.203
Borderline Features (BOR)	.986	2.085	.101
Antisocial Features (ANT)	.951	7.674	.000***
Alcohol Problems (ALC)	.972	4.279	.005**
Drug Problems (DRG)	.973	4.145	.006**
Aggression (AGG)	.975	3.742	.011*
Suicide Ideation (SUI)	.972	4.230	.006**
Stress (STR)	.986	2.176	.090
Non-Support (NON)	.998	.233	.874
Treatment Rejection (RXR)	.988	1.805	.145
Dominance (DOM)	.996	.629	.597
Warmth (WRM)	.994	.865	.459

Note.

***p < .001,

**p < .01,

*p < .05.

The discriminant analysis of the Model 1 (Age, Single/Widowed, Common law, Married, Separated/Divorced, Number of children, Years of education, Education, Employment status, Prior prison terms, and Total prison terms) and Model 2 (Model 1 + Psychological measures: SOM, ANX, ARD, DEP, MAN, PAR, SCZ, BOR, ANT, ALC, DRG, AGG, SUI, STR, NON, RXR, DOM, and WRM) incorporated all the measures of study in each case, not only those that showed discriminant capacity. Three discriminant functions were obtained in each model. The comparison between the values of the discriminant functions (referred to as Function 1) of both models shows that the relation of Model 2 to the four groups is somewhat stronger than that of Model 1. In any case, the canonical correlations of two discriminant functions are moderate (Table 5). Considering the standardized canonical discriminant function coefficients, Age (.638), Prior prison terms (-533), Total prison terms (-.303), SUI (.300), ANT (-.290), DRG (.217), SCZ (-.191), BOR (.152), Number of children (.137), STR (-.135), and Years of education (.132) are the measures of relatively greatest importance in the discriminant function of Model 2.

**Table 5:** Statistics of discriminant functions for models 1 and 2.

		Eigenvalue	Variance %	Canonical Correlation	Wilk’s Lambda	χ^2^	df
Model 1	Function 1	.332	70.7	.499	.658	185.157***	30
	Function 2	.100	21.3	.302	.876	58.380***	18
	Function 3	.037	7.9	.190	.964	16.168*	8
Model 2	Function 1	.400	56.2	.534	.536	270.400***	84
	Function 2	.197	27.7	.406	.750	124.768***	54
	Function 3	.115	16.1	.321	.897	46.961**	26

Note.

***p < .001,

**p < .01,

*p < .05;

df = Degrees of freedom; Model 1 = Actuarial data; Model 2 = Actuarial data + Psychological measures.

### Predictive accuracy of models

**Fig 1:**
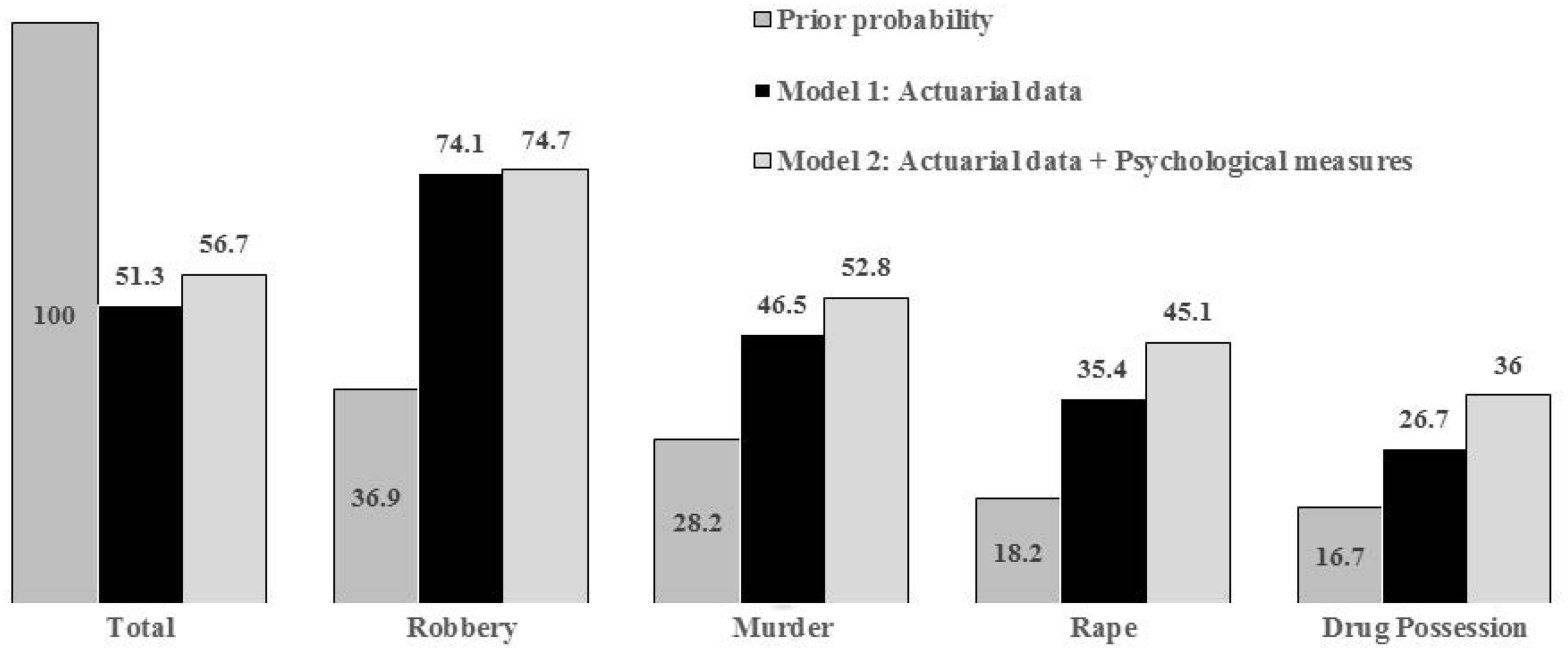
Percentages of correct classification for models 1 and 2.

By contrasting the degree of confidence in the predictions of each model, different results were recorded for each crime (Fig 1). Model 1 correctly classified 123 of the 166 convicted of Robbery, 59 of the 127 convicted of Murder, 29 of the 82 sentenced for Rape, and 20 of 75 convicted of Drug Possession. Model 2 correctly classified 124 of the 166 convicted of Robbery, 67 of the 127 sentenced for Murder, 37 of the 82 sentenced for Rape, and 27 of the 75 sentenced for Drug Possession. Fig 1 shows the previous percentages of classification according to the size of each group and percentage of correct classification (PCC) for each model.

### Distinct profile of each crime

Table 6 contains the correlations between the value of each measure and the probability of belonging to a group. Of all the explanatory variables, 5 SDM (Age, Common law, Married, Number of children, and Years of education), 2 JM (Prior prison terms and Total prison terms), and 6 PM (DEP, ANT, ALC, DRG, AGG, and SUI) met the established criteria (*r* >.25 and *p* < .001). The strength and sense of the contribution of these 13 correlations allowed us to construct four specific profiles. As a summary of the results, Table 7 facilitates the understanding of the characteristics that best differentiate each crime.

**Table 6:** Correlation between the value of each variable and the probability of belonging to a group.

Independent variable	Robbery	Murder	Rape	Drug Possession
Age	-.538***	-.126*	.432***	.496***
Single/Widowed	.185***	-.174***	.059	-.142**
Common law	-.019	.446***	-.240***	-.223***
Married	-.185***	-.319***	.159*	.469***
Separated/Divorced	-.047	-.140**	.140**	.083
Number of children	-.330***	.117*	.363***	-.017
Years of education	-.050	-.165***	-.014	.274***
Education	-.041	-.159**	-.005	.246***
Employment status	.091	-.090	.128**	-.168***
Prior prison terms	.678***	-.279***	-.578***	-.098*
Total prison terms	.612***	-.296***	-.399***	-.166***
Somatic Complaints (SOM)	-.060	.097*	.082	-.106*
Anxiety (ANX)	.046	.129**	-.030	-.183***
Anxiety-Related Disorders (ARD)	.086	.080	-.078	-.137**
Depression (DEP)	.030	.206***	-.015	-.260***
Mania (MAN)	.198***	-.231***	.041	-.078
Paranoia (PAR)	.185***	-.089	-.125**	-.046
Schizophrenia (SCZ)	.187***	-.072	-.011	-.188***
Borderline Features (BOR)	.204***	-.001	-.137**	-.162**
Antisocial Features (ANT)	.387***	-.323***	-.144**	-.065
Alcohol Problems (ALC)	.263***	-.325***	.052	-.083
Drug Problems (DRG)	.306***	-.259***	-.124**	-.037
Aggression (AGG)	.312***	-.277***	-.037	-.115*
Suicide Ideation (SUI)	-.114*	.093*	.262***	-.207***
Stress (STR)	.120*	.118*	-.078	-.232***
Non-Support (NON)	.080	-.099*	-.021	.014
Treatment Rejection (RXR)	-.134**	.113*	-.120*	.198***
Dominance (DOM)	-.059	-.079	.059	.115*
Warmth (WRM)	-.122**	.179***	-.089	.073

Note.

***p < .001,

**p < .01,

*p < .05;

Canonical load higher than *r* >.25 highlighted in bold.

**Table 7:** Summary of discriminant characteristics for crimes.

Crimes	Direction	Discriminant features	
		Actuarial data	Psychological measures
**Robbery**	+	Criminal records	ANT, ALC, DRG, AGG
	−	Age, Children	
**Murder**	+	Common law	
	−	Criminal records	ANT, ALC, DRG, AGG
**Rape**	+	Age, Children	SUI
	−	Criminal records	
**Drug Possession**	+	Age, Married, Years of study	
	−		DEP

*Note*. PAI scales: ANT = Antisocial Features; ALC = Alcohol Problems; DRG = Drug Problems; AGG = Aggression; SUI = Suicide Ideation; DEP = Depression.

## Discussion

The main objective of the present study was to analyze whether the discriminatory capacity of PM and AD varies according to the crime, using four groups of individuals convicted of common crimes. The analyses allowed us to determine the discriminating capacity and the classification accuracy of both types of measures within the same study and with the same sample. In addition, specific profiles of each crime were created based on their distinctive characteristics, for which the strongest significant correlations between the value of each explanatory variable and the probability of belonging to the group were considered. In short, it can be concluded that the findings provide support for the hypotheses. For a correct interpretation of the findings, it is important to remember that when categorizing the individuals by crime, any criminal proceedings in progress or previous convictions were not considered, and for each participant it was not possible to control the time that had elapsed between entering prison and the time of evaluation. Next, we will discuss the results regarding the classification accuracy of the models tested, the discriminating capacity of the PM and the AD, and the profile corresponding to each crime. Since the exhaustive assessment of the presence and contribution of each measure to identify a crime is beyond the scope of this study, we will mention the most noteworthy aspects of the structure of each profile.

### Classification accuracy of the models

The variance explained and the overall PCC of both models are quite acceptable. Indeed, the accuracy achieved by models 1 and 2, both generally and by crime, far exceeds the results that could be obtained by chance [39]. It is worth recalling that the purpose of this study is not to test a model that is supposed to have optimal classification accuracy, but to compare the usefulness of measures of a different nature to characterize the prison population by crimes. For this purpose, we used contrasting measures and those whose utility is assumed to be satisfactory in correctional settings [5,28,29].

### Discriminant capacity of psychological measures and actuarial data

The discriminant capacity analysis yields different results for both types of measures. The AD reveal superior discriminatory capacity with respect to PM, although their contribution varies substantially for each crime. The PCC of these measures obtains its maximum value for the crime of Robbery and progressively decreases for the three remaining crimes, reaching a discrepancy of almost 50% between the crimes of Robbery and Drug Possession. Instead, the PM reveal a different pattern and an inverse trend, as their PCC is insignificant in the case of Robbery, increases for Murder and reaches its highest values for the crimes of Rape and Drug Possession. These results appear to indicate that the importance given by the literature to socio-demographic data and that related to the individual’s criminal history [5,6,11,23,24,40,41] is consistent across different crimes. This contributes to the discriminatory strength of criminal records and age, particularly for Robbery, where the contribution of AD is almost exclusive.

In any case, the strength of the AD is not the same in the four crimes studied, which allows us to conclude that PM can enrich actuarial models, especially where they are less powerful. Support for this comes from the results obtained in PAI scales [32,33] that measure psychological constructs related to antisocial personality, substance abuse, depressive symptoms and suicidal ideation. It is reasonable, therefore, to expect that the classificatory diversity demonstrated by the PM and AD in four very frequent crimes worldwide [26] will occur in other crimes. To demonstrate this, it would be necessary to test more complete and rigorous designs in different populations of offenders. In this way, the measures that possess a high discriminatory power could be identified so that, when combined with those already mentioned in the literature, they form more accurate models for each crime. The classification and risk assessment approaches of various types of violence, useful for various intervention strategies, would benefit from the incorporation of these types of measures [5,23].

### Profile of Robbery crime

From the analysis of this profile it can be concluded that people convicted of Robbery tend to be younger, have fewer children, have more criminal records, possess more antisocial characteristics, are more aggressive and have more substance abuse problems. It is likely that the strength of this profile is due to the homogeneity of the group of individuals convicted of this crime and the discriminatory capacity of criminal records. The early onset of criminal activity, particularly in crimes against property [42,43], as well as the consistent relationship between age and violent recidivism [44] could explain the tendency of these individuals to accumulate a greater number of convictions for different crimes. In effect, this crime tends to be the result of general antisocial behavior and those who engage in robbery do so as part of a long line of offending behavior. In addition, the high frequency of individuals convicted of this crime in our study, particularly in the age range 18 to 30 years, is consistent with the trend observed worldwide [26]. On the other hand, the low average number of children could be explained by age and in terms of the theory of cumulative disadvantage [45], considering that the factors associated with criminal behavior during adolescence influence the adoption of a lifestyle that leads to poor social integration [46,47]. In addition, this profile is the only one where the ANT, ALC, DRG and AGG scales show moderate and direct loads. This corroborates the marked presence of antisocial characteristics, violent behavior, and substance abuse in this population [6,11,26,47].

### Profile of Murder crime

The findings appear to indicate that murderers are characterized by having a stable relationship, fewer criminal records, less antisocial characteristics, less aggressive behavior, and less substance abuse. In interpreting these results it should be considered that the contradictory data provided by the literature may be due to the complexity of the interaction between factors associated with homicide [48]. In this regard, the results appear to describe a more specific type of offender among individuals serving a sentence for murder. As for the first characteristic described, some authors point out the importance of marriage as a deterrent or attenuating factor in criminal activity in general [49–51]. Taking into account the components of the profile, and if we assume that having a partner has a status analogous to marriage in this population, the tendency of these individuals to have fewer criminal records can be explained. Within the various results provided by the literature, the remainder of our findings are consistent with those that suggest that murderers do not present more psychopathic characteristics and aggressiveness than other offenders [6,11,52]. Finally, although substance abuse is highly prevalent throughout the prison population [34,53], this problem does not appear to be one of the main characteristics of the killers.

### Profile of Rape crime

By way of a summary, it can be concluded that people convicted of rape tend to be older, have fewer criminal records, have more children, and present a higher level of suicidal ideation. This is incompatible, to a certain extent, with findings indicating that being young, being single, possessing certain antisocial characteristics of personality, and having a criminal record are factors that characterize a sex offender and contribute towards predicting their recidivism [24,54–58]. One explanation for this discrepancy is that subtypes of sex offender (e.g., rapist, abuser of children with and without paraphilia) may have unique defining characteristics [59–61]. This leads us to suppose that the subsample of sex offenders analyzed, which does not distinguish subtypes, could be composed mainly of people with a higher level of social integration, and who are unfamiliar with the delinquency and prison environment. In fact, this group of sex offenders does not reveal major psychopathological problems, particular those related to substance abuse that are common in other samples [54,59,61,62]. The presence of suicidal ideation, frequent in sexual aggressors, could be explained by the impact of incarceration, particularly for the first time, and aspects related to crime [62]. For those who lead an integrated social life (e.g., family, children, work), an abrupt change of context may represent a risk factor for various suicidal behaviors due to the loss of social references [63]. Other contributing factors in this regard are the expectations of a sentence, victimization by other inmates, and the feeling of guilt [62].

### Profile of Drug Possession crime

If we adhere to the factors that are part of this profile, those convicted of this crime appear to be characterized by being older, married, having a higher level of education and presenting fewer depressive symptoms. However, the reduced classification accuracy of the models for this crime and the scarce number of measures with relevant correlations make this profile the least strong of the four. Therefore, any interpretation must be taken with caution, and even more so if we consider that the heterogeneity of the subsample can account for the number and strength of the components of the profile. For example, it is not possible to determine whether the absence of criminal records in this group of offenders is truly a characteristic that identifies them or whether this is due to limitations of the model or variables that have not been controlled. Although we know that possession and drug trafficking are among the most persistent crimes from adolescence to adulthood [60], the factors that explain the late onset of crime have, however, not been clearly defined [64,65]. In this sense, the association between age, more years of study and marriage could explain the late onset of crime or a lower involvement in criminal activities compared with the other groups analyzed [48–51]. This level of social integration could reflect the provision of more individual resources and social support to cope with the conditions of life in prison. The tendency of these individuals to present less depressive symptoms and the absence of psychopathological indicators gives support to this interpretation.

### Strengths, limitations, and conclusions

This study compares, for the first time, the discriminant capacity and the classification accuracy of a broad set of psychological measures and actuarial data in a single study, with the same sample, and in several frequent crimes. In this context, this is one of the first works that uses the Spanish adaptation of the PAI, an ideal instrument for this type of approach because it allows for measuring the main variables of personality and psychopathology in a short time. However, it is reasonable to raise some concerns regarding the degree of understanding of Spanish used in the PAI questionnaire by the South American population. This supposed limitation was analyzed in the studies of linguistic adaptation of the Argentinian version of the PAI [66], where the content of only 4 of the 344 items that compose the PAI had to be modified to improve its comprehension. Other strengths to highlight are the sample size used, which allowed us to include a greater number of explanatory variables in the analysis, as well as the chosen crimes as opposed to categories of crimes.

One limitation of this study worth mentioning is the absence of important measures such as the crimes for which the sentence has been served in the past, the time of conviction for the current crime, gang membership, number of previous arrests, type and number of disciplinary infractions within prison and clinical and psychopathological antecedents before entering prison. Regarding the first six measures mentioned, we were not able to access this type of information. However, the participants did report their clinical and psychopathological antecedents, but the low frequency of these did not allow their inclusion in the analyses. Finally, it is likely that recording the previous convictions would have allowed for more sophisticated groups to be formed. We do not know, therefore, if this information had any impact on the accuracy of the results and subsequent construction of the profiles.

## Conclusions

In conclusion, the results of the present study indicate that the AD model is more robust when these four crimes are characterized; the contribution of AD and PM depends on the crime; and the inclusion of PM in actuarial models moderately optimizes the accuracy of classification in Murder, Rape and Drug Possession crimes. Specific profiles of each crime, composed of AD and PM, were also obtained, which demonstrates their usefulness in characterizing these crimes. These findings suggest the convenience of studying the prison population by crime, given that the latter have specific characteristics, using models that include the most relevant measures in each case. Future studies should analyze criminal profiles in a greater number of crimes and incorporate new measures, particularly those associated with violence [67,68].

## Supporting information

S1 File Full database (SPSS File). (ZIP)
